# Synthesis of 3-*O*- and 4-*O*-(2-aminoethylphosphono) derivatives of methyl l-*glycero*-α-d-*manno*-heptopyranoside

**DOI:** 10.1007/s00706-016-1868-6

**Published:** 2016-12-08

**Authors:** Martin Walter, Claudia Kohout, Markus Blaukopf, Paul Kosma

**Affiliations:** Department of Chemistry, University of Natural Resources and Life Sciences-Vienna, Vienna, Austria

**Keywords:** Lipopolysaccharide, Phosphorylations, Heptose, Carbohydrates, Glycosides

## Abstract

**Abstract:**

Phosphoethanolamine derivatives of the bacterial saccharide l-*glycero*-d-*manno*-heptose have been prepared using a phosphoramidite-based coupling reaction at position 4 of a side-chain-protected 2,3-*O*-orthoester methyl heptoside and at position 3 of a 3,4-diol heptoside, respectively. Global deprotection afforded the corresponding 2-aminoethylphosphodiester derivatives as substrates for crystallographic and binding studies with lectins and antibodies targeting the inner core structure of bacterial lipopolysaccharides.

**Graphical abstract:**

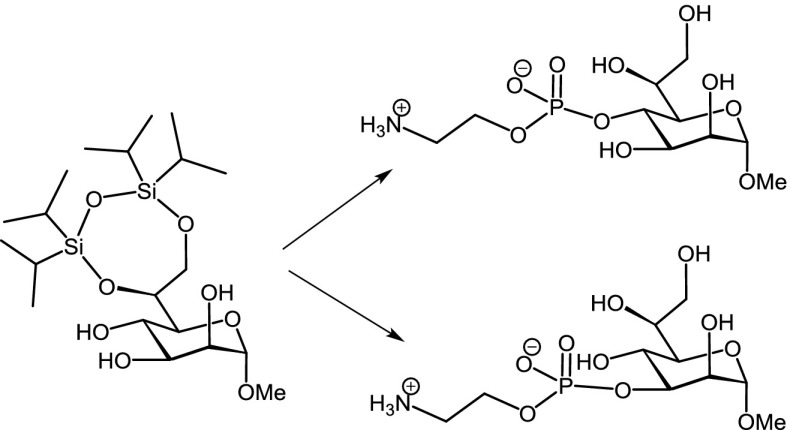

## Introduction

The outer membrane of the cell wall of Gram-negative bacteria harbors higher carbon sugars as characteristic components, which occur in the inner core region of bacterial lipopolysaccharides (LPS), but have also been detected in capsular polysaccharides (CPS) [1]. Heptoses of the l-*glycero*-d-*manno* configuration (L,D-Hep), in particular, constitute a structurally conserved domain in Enterobacteriaceae, such as in *Escherichia coli*, Salmonella or Yersinia, and are common LPS core determinants in the genera Haemophilus, Pseudomonas, Helicobacter, or Neisseria [2, 3]. Heptoses contribute toward many biomedically important interactions with the complement system, antibodies and lectins, and these features have been substantiated by recent data from crystallographic and glycan array studies [4–9]. These binding interactions and specificities are further modulated by additional phosphate substituents in the pyranose ring as well as at the exocyclic side chain.

Among the phosphate containing appendices, 2-aminoethyl phosphodiester (PEtn) groups have been found at positions 3, 4, 6, and 7 of L,D-Hep, and the group of Oscarson has successfully prepared 4-*O- *, 6-*O- *, and 7-*O-*substituted (2-aminoethyl)phosphate monoheptosides as well as various 3-*O- * and 6-*O*-PEtn substituted LPS oligosaccharides to unravel the structural basis for cross-reactive antibodies against *Neisseria meningitidis* and *Haemophilus influenzae*, respectively [10–13]. Recently, the structure of an antigen-binding fragment (Fab) from the bactericidal monoclonal antibody LPT3-1 complexed to an inner core octasaccharide fragment of *N. meningitidis* has been solved, which had been isolated via KOH treatment from the bacterial lipooligosaccharide [14]. The isolation protocol, however, leads to hydrolysis of the phosphoethanolamine units. As the 3-*O*-PEtn substituent is present in ~70% of *N. meningitidis* strains and constitutes a relevant epitope for the neutralizing antibodies, chemical synthesis is needed to provide material for binding and crystallographic studies [15]. For this purpose, we have set out to access both 3-*O*- and 4-*O*-substituted heptosides starting from a common intermediate with a minimum number of protecting group manipulations.

### Results and discussion

The previously reported 6,7-*O*-TBDPS protected heptoside **1** served as a versatile precursor for the introduction of the PEtn moiety via intermediate 2,3-orthoester formation as shown for the synthesis of 4-*O*-monophosphate derivatives [16]. In our hands, a three step sequence performed in a one-pot reaction could be elaborated to give a fair yield of the phosphotriester derivative **5** (Scheme 1). First, the reaction of **1** with *α*,*α*,*α*-triethoxytoluene (**2**) in the presence of camphorsulfonic acid (CSA) led to the intermediate orthoester **3**, which was followed by the application of the phosphoramidite procedure with [2-(benzyloxy-diisopropylamino-phosphanyloxy)ethyl]-carbamic acid benzyl ester (**4**) promoted by 1*H*-tetrazole, and the ensuing oxidation of the resulting phosphite with *meta*-chloroperbenzoic acid (*m*CPBA) [17–19]. Since the phosphorylated orthoester **5** was present as a mixture of four diastereoisomers, the product mixture was then separated into individual components to exclude the presence of potential impurities in the subsequent deprotection steps. MPLC separation allowed the isolation of a 1:3 mixture of the phosphorylated *endo* orthobenzoates **5a**, **5b** and *exo*-isomers **6a**, **6b** in 56% overall yield for three steps, followed by further HPLC separation of the phosphate diastereomers; no attempts, however, for assignment of the stereogenic center at phosphorus were undertaken. Assignment of the *exo*/*endo* configuration was based on the high-field shift of the *exo*-oriented OCH_2_ group at 3.30 ppm compared to the corresponding low-field shifted signal of the *endo*-isomer at 3.80 ppm [20].

**Figure:**
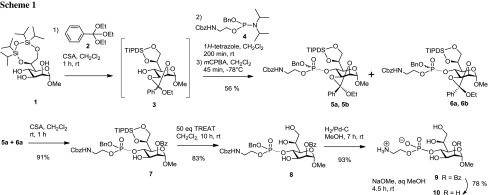

Next, the *endo*/*exo*-orthoester derivatives **5a** and **6a** (representing one of the diastereomeric forms on phosphorus) were subjected to acid-promoted orthoester opening, which produced the homogeneous 2-*O*-benzoyl derivative **7** in 91% yield. Compound **7** is equipped with an orthogonal protecting group pattern which allows access to chain elongation at position 3, as well as at the exocyclic side-chain positions. Removal of the 1,1,3,3-tetraisopropyl-1,3-disiloxane-1,3-diyl group was achieved by treatment of **7** with triethylamine trifluoride (TREAT). The reaction had to be monitored until full removal of the monofluorinated silyl intermediate (Ref. [16]) by TLC, to afford triol **8** in 83% yield. Hydrogenation of **8** was uneventful and gave the phosphodiester **9** in 93% yield. Cleavage of the benzoyl ester under Zemplén transesterification conditions was sluggish but eventually provided the 4-*O*-PEtn derivative **10** in good yield. Optical rotation values and ^13^C NMR data matched the previously reported data of **10**, which, however, had been synthesized via a different route based on *H*-phosphonate coupling chemistry [10].

The orthoester approach was then applied for the synthesis of the 3-*O*-substituted derivative **16** (Scheme 2). **1** was subjected to CSA-promoted orthoester formation with 1,1,1-trimethoxyethane to give 2,3-*O*-orthoacetate **11**, which was not isolated but directly converted into the 2-*O*-acetate **12** in 71% yield. The structure of ester **12** was readily assigned on the basis of the low-field shifted H-2 signal at 5.02 ppm. Based on previous evidence that a hydroxyl group adjacent to an axial one in a *cis*-vicinal diol is more reactive, and that the 4-OH group in a *manno*-pyranoside is much less reactive, a direct regioselective phosphorylation was expected to directly lead to the 3-*O*-substituted phosphoester, thereby avoiding additional protecting group manipulations [21, 22]. Thus, phosphorylation of diol **12** using **4** and 1*H*-tetrazole was followed by oxidation with *m*CPBA. The 3-*O*-substituted derivative **13** could then be separated from additional phosphorylated species by chromatography, and was isolated as a diastereomeric mixture in 32% yield.

**Figure:**
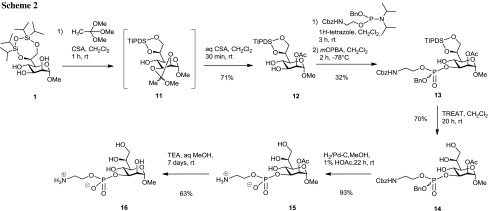

The structural assignment of **13** was based on the ^1^H-^31^P correlated HMBC spectrum, which showed the connectivity of H-3 to the phosphate unit, as well as on the low-field shifted H-3 signal at 4.21 ppm.

Similar to **6**, phosphotriester **13** was then treated with TREAT to give triol **14** in 70% yield, which was hydrogenated in the presence of 10% Pd–C in MeOH containing 1% acetic acid. The addition of the acid was needed to prevent formation of *N*-methylated products [23]. Removal of the 2-*O*-acetyl group was carried out by reaction of **15** with triethylamine in aqueous MeOH and furnished the deprotected zwitterionic glycoside **16** in 63% yield [24]. The structures of **10** and **16** were fully confirmed by their ^13^C NMR data, which showed a characteristic downfield shift of the respective carbons C-4 and C-3, respectively, involved in the phosphodiester linkage as well as heteronuclear *J*_C,P_ coupling interactions of C-4 and C-5 for **9** and C-3 and C-4 for **16** (Table 1). Similar *J*_C,P_ coupling interactions were also seen for the ethanolamine fragments. These assignments were further supported by ^1^H-^31^P HMBC connectivity data.

**Table 1:** ^13^C and ^31^P NMR chemical shifts (*δ*/ppm) and coupling constants (*J*/Hz) of **10** and **16** in D_2_O (27 °C)

Pos.	**10**		**16**	
	^13^C	*J*_C,P_	^13^C	*J*_C,P_
1	101.64	–	101.58	–
2	70.91	–	69.98	–
3	70.91	–	77.16	6.2
4	72.51	6.1	65.85	6.3
5	71.01	5.3	71.93	–
6	69.34	–	69.64	–
7	63.21	–	63.69	–
7′	–	–	–	–
PO*CH*_*2*_	63.13	5.5	62.81	n.d.
N*CH*_*2*_	40.87	7.8	40.89	7.2
OCH_3_	55.59	–	55.61	–

*n.d.* not determined

## Experimental Section

All purchased chemicals were used without further purification unless stated otherwise. Solvents were dried over activated 4 Å (CH_2_Cl_2_, pyridine) molecular sieves. Cation-exchange resin DOWEX 50 H^+^ was regenerated by consecutive washing with HCl (3 M), water, and dry MeOH. Aqueous solutions of salts were saturated unless stated otherwise. Concentration of organic solutions was performed under reduced pressure <40 °C. Optical rotations were measured with a Perkin-Elmer 243 B Polarimeter. Thin-layer chromatography was performed on Merck precoated plates: generally, on 5 × 10 cm, layer thickness 0.25 mm, silica gel 60F_254_; alternatively on HPTLC plates with 2.5 cm concentration zone (Merck). Spots were detected by dipping reagent (anisaldehyde-H_2_SO_4_). For column chromatography, silica gel (0.040–0.063 mm) was used. HP-column chromatography was performed on pre-packed columns (YMC-Pack SIL-06, 0.005 mm, 25 × 1 cm and 25 × 2 cm). NMR spectra were recorded with a Bruker Avance III 600 instrument (600.22 MHz for ^1^H, 150.93 MHz for ^13^C, and 242.97 MHz for ^31^P) using the standard Bruker NMR software. ^1^H spectra were referenced to 7.26 (CDCl_3_) and 0.00 (D_2_O, external calibration to 2,2-dimethyl-2-silapentane-5-sulfonic acid) ppm unless stated otherwise. ^13^C spectra were referenced to 77.00 (CDCl_3_), 49.00 (MeOD), and 67.40 (D_2_O, external calibration to 1,4-dioxane) ppm. ^31^P spectra were referenced to 0.00 ppm (orthophosphoric acid) for solutions in D_2_O and according to [25] for solutions in CDCl_3_. ESI–MS data were obtained on a Waters Micromass Q-TOF Ultima Global instrument.

### Methyl 2,3-*O*-(1-endo-ethoxybenzylidene)-4-*O*-[benzyl-2-(benzyloxycarbonylamino)ethylphosphono]-6,7-*O*-(1,1,3,3-tetraisopropyl-1,3-disiloxane-1,3-diyl)-l-glycero-α-d-manno-heptopyranoside (**5a**, **5b**, C_46_H_68_NO_14_PSi_2_) and methyl 2,3-*O*-(1-exo-ethoxybenzylidene)-4-*O*-[benzyl-2-(benzyloxycarbonylamino)ethylphosphono]-6,7-*O*-(1,1,3,3-tetraisopropyl-1,3-disiloxane-1,3-diyl)-l-glycero-α-d-manno-heptopyranoside (**6a**, **6b**, C_46_H_68_NO_14_PSi_2_)

Compound **1** (57.5 mg, 0.123 mmol) was coevaporated with dry toluene, and the residue was dissolved in 1.4 cm^3^ dry CH_2_Cl_2_ under Ar. *α*,*α*,*α*-Triethoxytoluene (33.5 mm^3^, 0.148 mmol) and 1.2 mg solid camphor sulfonic acid (6 μmol) were then added at rt and the solution was stirred for 1 h. Triethylamine (21.8 mm^3^, 0.156 mmol) was then added, the solution was concentrated, and the residue was coevaporated with toluene. The residue was dissolved in 1.4 cm^3^ dry CH_2_Cl_2_, and 79.3 mm^3^ phosphoramidite reagent **4** (0.18 mmol) was added followed by dropwise addition of 0.36 cm^3^ (of a 0.45 M solution of 1*H*-tetrazole in acetonitrile. Additional amounts of **4** (4 × 30 mm^3^, 0.277 mmol) and the 0.45 M solution of 1*H*-tetrazole in acetonitrile (1 × 0.17 cm^3^ and 3 × 0.13 cm^3^, 0.269 mmol) were added in four portions over 200 min. The reaction mixture was then cooled to −78 °C, and a solution of 166 mg *meta*-chloroperbenzoic acid (77% content, 0.739 mmol) in 1.5 cm^3^ dry CH_2_Cl_2_ was added and stirred at −78 °C for 45 min. TEA (0.137 cm^3^) was then added, and the mixture allowed to warm up to rt. The reaction mixture was then transferred into a two-phase solution of aqueous NaHCO_3_ and EtOAc (each 20 cm^3^) followed by phase separation. The aqueous phase was extracted once more with 10 cm^3^ EtOAc. The combined organic phases were washed with brine, dried (Na_2_SO_4_), and concentrated. The residue was subjected to chromatography on silica gel (2 g, hexane/EtOAc = 3/1 → 2.5/1, containing 0.5% TEA) which gave a product fraction (67 mg) and a fraction containing byproducts (49 mg). The latter fraction was rechromatographed to afford an additional amount of product (25.9 mg). The combined product fractions were then submitted to MPLC separation (hexane/EtOAc = 3.5/1, containing 0.3% TEA) which afforded a mixture of *endo*-orthoester **5a** and **5b** (16.1 mg, 14%) and *exo*-orthoester **6a** and **6b** (49.7 mg, 42%) as oils. Both fractions were further separated by HPLC (hexane/EtOAc = 3/1, containing 0.3% TEA) which gave *endo*-isomer **5a** (5.0 mg) and *endo*-isomer **5b**; *R*_f_ = 0.57 (hexane/EtOAc = 1:1); oil.

^1^H NMR (600 MHz, CDCl_3_) for **5a**: *δ* = 7.52–7.48 (m, 2H, H^Ar^), 7.38–7.35 (m, 2H, H^Ar^), 7.35–7.26 (m, 11H, H^Ar^), 5.40 (bs, 1H, NH), 5.14 (dd, *J*_H,P_ = 7.8 Hz, *J* = 11.9 Hz, 1H, POC*H*_2_Ph), 5.07 (dd, *J*_H,P_ = 9.2 Hz, *J* = 11.9 Hz, 1H, POC*H*_2_Ph), 5.03–5.00 (m, 2H, C*H*_2_Ph), 5.00 (ddd, *J*_H,P_ = 8.9 Hz, *J* = 6.9, 10.0 Hz, 1H, H-4), 4.94 (s, 1H, H-1), 4.54 (app t, *J* = *J* = 6.9 Hz, 1H, H-3), 4.35 (app d, *J* = 8.7 Hz, 1H, H-6), 4.13 (dd, *J* = 8.8, *J* = 12.2 Hz, 1H, H-7a), 4.17–4.05 (m, 2H, NCH_2_C*H*_2_OP), 3.98 (dd, *J* = 0.8, *J* = 6.8 Hz, 1H, H-2), 3.86 (dd, *J* = 1.4, *J* = 12.2 Hz, 1H, H-7b), 3.82–3.73 (m, 2H, OC*H*_2_CH_3_), 3.65 (dd, *J* = 1.4, *J* = 10.1 Hz, 1H, H-5), 3.46–3.40 (m, 2H, C*H*_*2*_N), 3.37 (s, 3H, OCH_3_), 1.17 (t, *J* = 7.1 Hz, 3H, OCH_2_C*H*_3_), 1.12–0.92 (m, 28H, TIPDS) ppm; ^13^C NMR (151 MHz, CDCl_3_): *δ* = 138.8 (C-1^Ar^), 129.0, 128.6, 128.4, 128.2, 128.1, 128.0, 127.9, 125.9 (C^Ar^), 121.8 (Cq, orthoester), 98.3 (C-1), 76.6 (C-3), 75.4 (C-2), 74.9 (d, *J*_C,P_ = 5.5 Hz, C-4), 73.6 (C-6), 69.6 (d, *J*_C,P_ = 5.5 Hz, POCH_2_), 68.9 (d, *J*_C,P_ = 9.9 Hz, C-5), 68.3 (C-7), 67.2 (d, *J*_C,P_ = 5.5 Hz, NCH_2_ *C*H_2_OP), 66.7 (*C*H_2_Ph), 59.2 (O*C*H_2_CH_3_), 55.4 (OCH_3_), 41.5 (d, *J*_C,P_ = 7.7 Hz, N*C*H_2_), 17.7, 17.6, 17.4, 17.34, 17.28, 17.25, 17.2 (8 × TIPDS-*C*H_3_), 14.9 (OCH_2_ *C*H_3_), 13.33, 13.30, 12.7, 12.6 (4 × TIPDS-*C*H) ppm; ^31^P NMR (243 MHz, CDCl_3_): *δ* = −2.36 ppm.

^1^H NMR (600 MHz, CDCl_3_) for **5b**: *δ* = 7.48–7.45 (m, 2H, H^Ar^), 7.39–7.36 (m, 2H, H^Ar^), 7.36–7.26 (m, 11H, H^Ar^), 5.33 (bs, 1H, NH), 5.17 (dd, *J*_H,P_ = 8.0 Hz, *J* = 11.9 Hz, 1H, POC*H*_2_Ph), 5.14 (dd, *J*_H,P_ = 9.3 Hz, *J* = 11.8 Hz, 1H, POC*H*_2_Ph), 5.07–5.02 (m, 2H, C*H*_2_Ph), 4.99 (app dt, *J*_H,P_ = 7.0 Hz, *J* = 9.6 Hz, 1H, H-4), 4.95 (s, 1H, H-1), 4.62 (app t, *J* = 7.1 Hz, 1H, H-3), 4.33 (app d, *J* = 8.7 Hz, 1H, H-6), 4.13 (dd, *J* = 8.6, *J* = 12.2 Hz, 1H, H-7a), 4.10–4.04 (m, 2H, NCH_2_C*H*_2_OP), 3.98 (d, *J* = 6.9 Hz, 1H, H-2), 3.86 (app d, *J* = 12.1 Hz, 1H, H-7b), 3.81–3.75 (OC*H*_2_CH_3_), 3.68 (dd, *J* = 1.3, *J* = 9.9 Hz, 1H, H-5), 3.47–3.35 (m, 2H, C*H*_*2*_N), 3.34 (s, 3H, OCH_3_), 1.18 (t, *J* = 7.1 Hz, 3H, CH_3_), 1.13–0.93 (m, 28H, TIPDS) ppm; ^13^C NMR (151 MHz, CDCl_3_): *δ* = 138.8 (C-1^Ar^), 136.5 (C-1^Ar^), 135.9 (d, *J*_C,P_ = 6.6 Hz, C-1^Ar^), 129.0, 128.6, 128.6, 128.5, 128.2, 128.1, 128.0, 127.9, 125.9 (C^Ar^), 121.8 (Cq, orthoester), 98.3 (C-1), 76.4 (C-3), 75.5 (C-2), 75.0 (d, *J*_C,P_ = 6.1 Hz, C-4), 73.6 (C-6), 69.8 (d, *J*_C,P_ = 5.3 Hz, PO*C*H_2_), 68.9 (d, *J*_C,P_ = 9.9 Hz, C-5), 68.3 (C-7), 67.0 (d, *J*_C,P_ = 5.5 Hz, NCH_2_ *C*H_2_OP), 66.7 (*C*H_2_Ph), 59.2 (O*C*H_2_CH_3_), 55.4 (OCH_3_), 41.4 (d, *J*_C,P_ = 7.7 Hz, N*C*H_2_), 17.7, 17.6, 17.4, 17.34, 17.27, 17.26, 17.2 (8 × TIPDS-*C*H_3_),15.0 (OCH_2_ *C*H_3_), 13.3, 13.2, 12.7, 12.6 (4 × TIPDS-*C*H) ppm; ^31^P NMR (243 MHz, CDCl_3_): *δ* = −2.52 ppm.

HPLC separation (hexane/EtOAc = 2.5/1) of the *exo*-orthoester fraction afforded *exo*-isomer **6a** (22.7 mg) and *exo*-isomer **6b** (18.5 mg); *R*_f_ = 0.45 (hexane/EtOAc = 1/1); oil.

^1^H NMR (600 MHz, CDCl_3_) for **6a**: *δ* = 7.66–7.61 (m, 2H, H^Ar^), 7.35–7.28 (m, 13H, H^Ar^), 5.41 (bs, 1H, NH), 5.08–5.04 (m, 3H, 2 × C*H*_2_Ph, POC*H*_2_Ph), 5.00 (s, 1H, H-1), 4.91 (dd, *J*_H,P_ = 9.5, *J* = 11.8 Hz, 1H, POC*H*_2_Ph), 4.76 (app t, *J* = 6.6 Hz, 1H, H-3), 4.50 (app dt, *J*_H,P_ = 6.8, *J* = 9.7 Hz, 1H, H-4), 4.49 (d, *J* = 6.7 Hz, 1H, H-2), 4.26 (app d, *J* = 8.7 Hz, 1H, H-6), 4.07 (dd, *J* = 8.7, 12.2 Hz, 1H, H-7a), 4.04–3.93 (m, 2H, NCH_2_C*H*_2_OP), 3.81 (dd, *J* = 1.4, *J* = 12.1 Hz, 1H, H-7b), 3.62 (dd, *J* = 1.5, *J* = 9.9 Hz, 1H, H-5), 3.37 (s, 3H, OCH_3_), 3.41–3.27 (m, 4H, CH_2_N, OC*H*_2_CH_3_), 1.10 (t, *J* = 7.05 Hz, 3H, CH_3_), 1.08–0.79 (m, 28H, TIPDS) ppm; ^13^C NMR (151 MHz, CDCl_3_): *δ* = 156.4 (NC=O), 136.7 (C-1^Ar^), 136.5 (C-1^Ar^), 135.7 (d, *J* = 6.6 Hz, C-1^Ar^), 129.2, 128.6, 128.4, 128.2, 128.1, 128.0, 127.9, 126.4 (C^Ar^), 120.9 (Cq, orthoester), 98.0 (C-1), 77.5 (C-3), 75.5 (C-2), 75.1 (d, *J*_C,P_ = 6.6 Hz, C-4), 73.3 (C-6), 69.5 (d, *J*_C,P_ = 5.5 Hz, PO*C*H_2_), 68.5 (d, *J*_C,P_ = 9.9 Hz, C-5), 68.1 (C-7), 67.1 (d, *J* = 5.5 Hz, NCH_2_ *C*H_2_OP), 66.7 (*C*H_2_Ph), 59.5 (O*C*H_2_CH_3_), 55.4 (OCH_3_), 41.4 (d, *J*_C,P_ = 5.5 Hz, N*C*H_2_), 17.6, 17.5, 17.4, 17.3, 17.24, 17.21, 17.1 (8 × TIPDS-*C*H_3_), 15.1 (OCH_2_ *C*H_3_), 13.3, 13.2, 12.7, 12.6 (4 × TIPDS-*C*H) ppm; ^31^P NMR (243 MHz, CDCl_3_): *δ* = −2.58 ppm.

^1^H NMR (600 MHz, CDCl_3_) for **6b**: *δ* = 7.67–7.62 (m, 2H, H^Ar^), 7.36–7.27 (m, 13H, H^Ar^), 5.29 (bs, 1H, NH), 5.07 (s, 2H, C*H*_2_Ph), 5.04 (dd, *J*_H,P_ = 8.0, *J* = 11.9 Hz, 1H, POC*H*_2_Ph), 5.00 (s, 1H, H-1), 4.99 (dd, *J*_H,P_ = 9.6, *J* = 11.9 Hz, 1H, POC*H*_2_Ph), 4.83 (app t, *J* = 6.7 Hz, 1H, H-3), 4.50 (d, *J* = 6.2 Hz, 1H, H-2), 4.46 (app dt, *J*_H,P_ = 6.9, *J* = 10.3 Hz, 1H, H-4), 4.26 (app d, *J* = 8.7 Hz, 1H, H-6), 4.07 (dd, *J* = 8.9, *J* = 12.2 Hz, 1H, H-7a), 4.00–3.91 (m, 2H, NCH_2_C*H*_2_OP), 3.81 (dd, *J* = 1.4, *J* = 12.2 Hz, 1H, H-7b), 3.68 (dd, *J* = 1.5, *J* = 9.9 Hz, 1H, H-5), 3.37 (s, 3H, OCH_3_), 3.39–3.25 (m, 4H, CH_2_N, OC*H*_2_CH_3_), 1.10 (t, *J* = 7.0 Hz, 3H, C*H*_3_), 1.08–0.78 (m, 28H, TIPDS) ppm; ^13^C NMR (151 MHz, CDCl_3_): *δ* = 156.3 (NC=O), 136.9 (C-1^Ar^), 136.5 (C-1^Ar^), 135.8 (d, *J*_C,P_ = 6.3 Hz, C-1^Ar^), 129.1, 128.54, 128.53, 128.46, 128.2, 128.08, 128.06, 127.9, 126.4 (C^Ar^), 120.8 (Cq, orthoester), 98.0 (C-1), 77.3 (C-3), 75.7 (C-2), 75.4 (d, *J*_C,P_ = 6.6 Hz, C-4), 73.3 (C-6), 69.7 (d, *J*_C,P_ = 5.5 Hz, POCH_2_), 68.5 (d, *J*_C,P_ = 8.8 Hz, C-5), 68.0 (C-7), 67.0 (d, *J*_C,P_ = 5.5 Hz, NCH_2_ *C*H_2_OP), 66.7 (*C*H_2_Ph), 59.4 (O*C*H_2_CH_3_), 55.4 (OCH_3_), 41.3 (d, *J*_C,P_ = 7.7 Hz, N*C*H_2_), 17.5, 17.43, 17.38, 17.3, 17.24, 17.21, 17.1 (8 × TIPDS-*C*H_3_), 15.1 (OCH_2_ *C*H_3_), 13.2, 13.1, 12.7, 12.6 (4 × TIPDS-*C*H) ppm; ^31^P NMR (243 MHz, CDCl_3_): *δ* = −3.07 ppm; HRMS (^+^ESI-TOF): *m/z* calcd for C_46_H_68_NO_14_PSi_2_ ([M+Na]^+^) 968.3808, found 968.3822.

### Methyl 2-*O*-benzoyl-4-*O*-[benzyl-2-(benzyloxycarbonylamino)ethylphosphono]-6,7-*O*-(1,1,3,3-tetraisopropyl-1,3-disiloxane-1,3-diyl)-l-glycero-α-d-manno-heptopyranoside (**7**, C_44_H_64_NO_14_PSi_2_)

A solution of *exo/endo*-orthoester **5a** and **6a** (26 mg, 27.5 μmol) in 2.6 cm^3^ CH_2_Cl_2_ was stirred with 0.64 mg camphorsulfonic acid (2.75 μmol) for 1 h at rt. Triethylamine (3.8 mm^3^) was added, and the mixture was concentrated. The residue was purified by chromatography on silica gel (hexane/EtOAc = 2/1 → 3/1) which gave 22.9 mg (91%) **7** as colorless oil; *R*_f_ = 0.26 (hexane/EtOAc = 1/1); [*α*]_D_^20^ = +27.3° cm^2^ g^−1^ (*c* = 1.8, CHCl_3_); ^1^H NMR (600 MHz, CDCl_3_): *δ* = 8.05–8.00 (m, 2H, H-2/H-6^Ar^), 7.56–7.51 (m, 1H, H-4^Ar^), 7.41–7.37 (m, 2H, H-3/H-5^Ar^), 7.37–7.27 (m, 10H, H^Ar^), 5.37 (dd, *J* = 1.6, 3.6 Hz, 1H, H-2), 5.28 (bs, 1H, NH), 5.10 (d, *J*_H,P_ = 8.9 Hz, 2H, POC*H*_2_Ph), 5.03 (d, *J* = 12.3 Hz, 1H, C*H*_2_Ph), 4.97 (d, *J* = 12.3 Hz, 1H, C*H*_2_Ph), 4.79 (d, *J* = 1.5 Hz, 1H, H-1), 4.78 (app q, *J* = 9.3 Hz, 1H, H-4), 4.43 (d, *J* = 5.3 Hz, 1H, 3-OH), 4.35 (ddd, *J* = 3.9, *J* = 5.3, *J* = 9.4 Hz, 1H, H-3), 4.31 (app d, *J* = 8.6 Hz, 1H, H-6), 4.13–4.04 (m, 3H, H-7a, NCH_2_C*H*_2_OP), 3.80 (dd, *J* = 1.1, *J* = 12.4 Hz, 1H, H-7b), 3.62 (dd, *J* = 1.1, *J* = 9.6 Hz, 1H, H-5), 3.47–3.40 (m, 1H, CH_2_N), 3.40–3.32 (m, 1H, CH_2_N), 3.34 (s, 3H, OCH_3_), 1.15–0.83 (m, 28H, TIPDS) ppm; ^13^C NMR (151 MHz, CDCl_3_): *δ* = 165.8 (C=O), 156.3 (NC=O), 136.4 (C-1^Ar^), 135.4 (d, *J*_C,P_ = 6.7 Hz, C-1^Ar^), 133.3 (C-4^Ar^), 129.9 (C-2/C-6^Ar^), 129.6 (C-1^Ar^), 128.8, 128.68, 128.67, 128.5, 128.3, 128.03, 128.01, 127.97 (C^Ar^), 98.4 (C-1), 76.9 (d, *J*_C,P_ = 6.6 Hz, C-4), 73.4 (C-6), 72.4 (C-2), 71.0 (d, *J*_C,P_ = 9.5 Hz, C-5), 70.1 (d, *J*_C,P_ = 5.5 Hz, POCH_2_), 68.4 (C-3), 68.1 (C-7), 67.6 (d, *J*_C,P_ = 5.4 Hz, NCH_2_ *C*H_2_OP), 66.7 (*C*H_2_Ph), 55.2 (OCH_3_), 41.3 (d, *J*_C,P_ = 5.5 Hz, N*C*H_2_), 17.6, 17.5, 17.34, 17.33, 17.30, 17.25, 17.2, 17.1 (8 × TIPDS-*C*H_3_), 13.4, 13.3, 12.6 (4 × TIPDS-*C*H) ppm; ^31^P NMR (243 MHz, CDCl_3_): *δ* = −0.38 ppm; HRMS (^+^ESI-TOF): *m/z* calcd for C_44_H_65_NO_14_PSi_2_ ([M+H]^+^) 918.3676, found 918.3673.

### Methyl 2-*O*-benzoyl-4-*O*-[benzyl-2-(benzyloxycarbonylamino)ethylphosphono]-l-glycero-α-d-manno-heptopyranoside (**8**, C_32_H_38_NO_13_P)

A solution of 20.5 mg **7** (22.3 μmol) in 1 cm^3^ CH_2_Cl_2_ was transferred to a Teflon-flask and cooled to ice-bath temperature. TREAT (73 mm^3^, 0.45 μmol) was added, and the solution was vigorously stirred at rt. Additional portions of TREAT (2 × 27 μcm^3^, 0.17 μmol) were added after 4 and 7.5 h reaction time. After 10 h, the solution was transferred into an ice-cold solution of aqueous NaHCO_3_ (5 cm^3^) followed by extraction with 10 cm^3^ portions of EtOAc. The combined organic layer was washed with brine, dried (Na_2_SO_4_), and concentrated. The crude residue (17.4 mg) was purified by chromatography on a Biotage Isolute Flash Si II column using hexane/EtOAc = 1/2 → EtOAc for elution, which afforded 11.5 mg (83%) **8** as colorless syrup. *R*_f_ = 0.19 (EtOAc); [*α*]_D_^20^ = +0.3° cm^2^ g^−1^ (*c* = 1.25, CHCl_3_); ^1^H NMR (600 MHz, CDCl_3_): *δ* = 8.05–8.02 (m, 2H, H-2/H-6 ^Ar^), 7.58–7.54 (m, 1H, H-4^Ar^), 7.44–7.40 (m, 2H, H-3/H-5^Ar^), 7.40–7.27 (m, 10H, H^Ar^), 5.42 (bs, 1H, NH), 5.37 (dd, *J* = 1.6, *J* = 3.6 Hz, 1H, H-2), 5.13 (d, *J*_H,P_ = 8.8 Hz, 2H, POC*H*_2_Ph), 5.01 (d, *J* = 12.1 Hz, 1H, C*H*_2_Ph), 4.98 (d, *J* = 12.1 Hz, 1H, C*H*_2_Ph), 4.84 (br s, 1H, H-1), 4.79 (app q, *J* = 9.7 Hz, 1H, H-4), 4.27 (ddd, *J* = 3.7, *J* = 5.7, *J* = 9.4 Hz, 1H, H-3), 4.15–4.04 (m, 2H, NCH_2_C*H*_2_OP), 3.99–3.95 (m, 1H, H-6), 3.85 (dd, *J* = 7.5, *J* = 10.8 Hz, 1H, H-7a), 3.73 (br d, *J* = 9.8 Hz, 1H, H-5), 3.66–3.59 (m, 1H, H-7b), 3.53 (bs, 1H, 3-OH), 3.47 (bs, 1H, 6-OH), 3.42–3.36 (m, 2H, CH_2_N), 3.37 (s, 3H, OCH_3_), 2.16 (bs, 1H, 7-OH) ppm; ^13^C NMR (151 MHz, CDCl_3_): *δ* = 166.0 (C=O), 156.5 (NC=O), 136.3 (C-1^Ar^), 135.2 (d, *J*_C,P_ = 6.6 Hz, C1^Ar^), 133.5 (C-4^Ar^), 129.9 (C-2/C-6^Ar^), 129.3 (C-1^Ar^), 128.9, 128.7, 128.5, 128.5, 128.12, 128.05, 128.0 (C^Ar^), 98.7 (C-1), 74.9 (d, *J*_C,P_ = 5.5 Hz, C-4), 72.4 (C-2), 70.4 (d, *J*_C,P_ = 6.1 Hz, C-5), 70.3 (d, *J*_C,P_ = 5.5 Hz, POCH_2_), 68.7 (d, *J*_C,P_ = 2.6 Hz, C-3), 68.4 (C-6), 67.5 (d, *J*_C,P_ = 6.3 Hz, NCH_2_ *C*H_2_OP), 66.8 (*C*H_2_Ph), 63.5 (C-7), 55.4 (OCH_3_), 41.1 (d, *J*_C,P_ = 6.6 Hz, N*C*H_2_) ppm; ^31^P NMR (243 MHz, CDCl_3_): *δ* = 0.24 ppm; HRMS (^+^ESI-TOF): *m/z* calcd for C_32_H_39_NO_13_P ([M+H]^+^) 676.2154, found 676.2164.

### Methyl 4-*O*-(2-aminoethylphosphono)-2-*O*-benzoyl-l-glycero-α-d-manno-heptopyranoside (**9**, C_17_H_26_NO_11_P)

A suspension of 20.0 mg **8** (0.03 mmol) and 4.6 mg Pd–C (10%) in 2 cm^3^ MeOH was hydrogenated at atmospheric pressure for 7 h at rt. The suspension was filtered over Celite® and thoroughly rinsed with MeOH. The filtrate was concentrated to give 12.4 mg (93%) **9** as amorphous glass.

*R*_f_ = 0.64 (CHCl_3_/MeOH/H_2_O = 5/4/1); [*α*]_D_^20^ = +8.4° cm^2^ g^−1^ (*c* = 1.25, MeOH); ^1^H NMR (600 MHz, MeOD): *δ* = 8.13–8.08 (m, 2H, H-2/H-6^Ar^), 7.64–7.59 (m, 1H, H-4^Ar^), 7.51–7.46 (m, 2H, H-3/H-5^Ar^), 5.30 (dd, *J* = 1.2, 3.2 Hz, 1H, H-2), 4.79 (br s, 1H, H-1), 4.61 (app q, *J* = 9.5 Hz, 1H, H-4), 4.21–4.15 (m, 2H, H-3, C*H*_2_OP), 4.15–4.08 (m, 2H, H-6, C*H*_2_OP), 3.77 (app d, *J* = 9.2 Hz, 1H, H-5), 3.75 (dd, *J* = 6.9, *J* = 10.5 Hz, 1H, H-7a), 3.69 (dd, *J* = 6.6, *J* = 10.7 Hz, 1H, H-7b), 3.41 (s, 3H, OCH_3_), 3.19–3.11 (m, 2H, CH_2_N) ppm; ^13^C NMR (151 MHz, MeOD): *δ* = 167.4 (C=O), 134.5 (C-4^Ar^), 131.2 (C-1^Ar^), 131.0 (C-2/C-6^Ar^), 129.5 (C-3/C-5^Ar^), 100.1 (C-1), 74.1 (C-2), 73.2 (d, *J*_C,P_ = 5.8 Hz, C-4), 71.8 (d, *J*_C,P_ = 5.5 Hz, C-5), 70.7 (C-3), 70.03 (C-6), 63.5 (C-7), 63.2 (d, *J*_C,P_ = 5.3 Hz, CH_2_OP), 55.6 (OCH_3_), 41.5 (d, *J*_C,P_ = 7.7 Hz, CH_2_N) ppm; ^31^P NMR (243 MHz, D_2_O): *δ* = 0.99 ppm; HRMS (^+^ESI-TOF): *m/z* calcd for C_17_H_27_ NO_11_P ([M+H]^+^) 452.1316, found 452.1323.

### Methyl 4-*O*-(2-aminoethylphosphono)-l-glycero-α-d-manno-heptopyranoside (**10**, C_10_H_21_NO_10_P)

A solution of 11.8 mg **9** (26 μmol) was coevaporated with toluene. The residue was dissolved in 1.2 cm^3^ dry MeOH and stirred with 0.1 cm^3^ 1 M methanolic NaOMe for 4.5 h at rt. The solution was made neutral by the addition of Dowex 50 cation-exchange resin (H^+^-form). The suspension was filtered, and the filtrate was concentrated. The residue was dissolved in 1 cm^3^ D_2_O and extracted three times with 1.5 cm^3^ portions of diethylether. The combined organic phase was re-extracted with 0.7 cm^3^ D_2_O, filtered over glass-wool, and purged with argon to remove residual ether. The aqueous phase was lyophilized to give 7.1 mg (78%) **10** as amorphous solid. *R*_f_ = 0.17 (CHCl_3_/MeOH/H_2_O = 5/4/1); [*α*]_D_^20^ = +43° cm^2^ g^−1^ (*c* = 0.7, H_2_O) (Ref. [10]: [*α*]_D_^20^ = +37° cm^2^ g^−1^ (*c* = 1.0, H_2_O)); ^1^H NMR (600 MHz, D_2_O, pD ~7.5 to 8.0): *δ* = 4.74 (d, *J* = 1.5 Hz, 1H, H-1), 4.31 (app q, *J* = 9.5 Hz, 1H, H-4), 4.19–4.10 (m, 2H, CH_2_OP), 4.04 (ddd, *J* = 1.3, *J* = 5.4, *J* = 7.6 Hz, 1H, H-6), 3.92 (dd, *J* = 1.7, *J* = 3.5 Hz, 1H, H-2), 3.89 (dd, *J* = 3.5, *J* = 9.2 Hz, 1H, H-3), 3.74 (dd, *J* = 7.6, *J* = 11.3 Hz, 1H, H-7a), 3.70 (dd, *J* = 5.4, *J* = 11.3 Hz, 1H, H-7b), 3.67 (dd, *J* = 1.2, *J* = 9.8 Hz, 1H, H-5), 3.35 (s, 3H, OCH_3_), 3.27–3.21 (m, 2H, C*H*_*2*_N) ppm; ^13^C NMR (151 MHz, D_2_O): see Table 1; ^31^P NMR (243 MHz, D_2_O): *δ* = 0.35 ppm; HRMS (^−^ESI-TOF): *m/z* calcd for C_10_H_20_NO_10_P ([M−H]^−^) 346.0909, found 346.0906.

### Methyl 2-*O*-acetyl-6,7-*O*-(1,1,3,3-tetraisopropyl-1,3-disiloxane-1,3-diyl)-l-glycero-α-d-manno-heptopyranoside (**12**, C_22_H_44_O_9_Si_2_)

1,1,1-Trimethoxyethane (35 mg, 0.291 mmol) and 3 mg camphorsulfonic acid (0.012 mmol) were added to a solution of 113 mg heptoside **1** (0.242 mmol) in 1 cm^3^ dry CH_2_Cl_2_ and stirred for 1 h at rt when TLC showed full conversion of **1** into intermediate **11** (*R*_f_ = 0.33, hexane/EtOAc = 2/1). Water (0.01 cm^3^) and triethylamine (0.01 cm^3^) were then added, and stirring was continued for 30 min at rt. The solution was diluted with CH_2_Cl_2_, and the organic layer was extracted with aqueous NaHCO_3_ and brine. The organic phase was dried (Na_2_SO_4_) and concentrated, and the residue was purified via flash chromatography (Isolute Flash Si II 2 g/6 cm^3^, hexane/EtOAc = 2/1) and by HPLC (column YMC-Pack-Sil-06 250 × 20 mm, hexane/EtOAc = 4/1 → 2/1) to give 88 mg (71%) **12** as syrup. *R*_f_ = 0.59 (hexane/EtOAc = 1/1); [*α*]_D_^21^ = +22.6° cm^2^ g^−1^ (*c* = 1.2, CHCl_3_); ^1^H NMR (600 MHz, CDCl_3_): *δ* = 5.02 (dd, 1H, *J* = 3.3, *J* = 1.7 Hz, H-2), 4.69 (d, 1H, *J* = 1.6 Hz, H-1), 4.33–4.30 (m, 1H, H-6), 4.06–4.02 (m, 1H, H-7a), 4.00–3.91 (m, 3H, H-3, H-4, H-7b), 3.57 (dd, 1H, *J* = 2.5, *J* = 9.2 Hz, H-5), 3.33 (s, 3H, OCH_3_), 2.10 (s, 3H, OAc), 1.13–0.93 (m, 28H, TIPDS) ppm; ^13^C NMR (151 MHz, CDCl_3_): *δ* = 170.76 (C=O), 98.5 (C-1), 74.7 (C-6), 71.8 (C-2), 71.65 (C-5), 70.4 (C-3), 67.9 (C-4), 67.6 (C-7), 55.0 (OCH_3_), 20.85 (OAc), 17.5, 17.4, 17.36, 17.34, 17.28, 17.27, 17.22, 13.1, 12.7 (d.i.), 12.4 (TIPDS) ppm; HRMS (ESI): *m/z* calcd for C_22_H_44_O_9_Si_2_ + H^+^ ([M+H]^+^) 509.2597, found 509.2614.

### Methyl 2-*O*-acetyl-3-*O*-[benzyl-(2-N-benzyloxycarbonylaminoethyl)phosphono]-6,7-*O*-(1,1,3,3-tetraisopropyl-1,3-disiloxane-1,3-diyl)-l-glycero-α-d-manno-heptopyranoside (**13**, C_39_H_62_NO_14_PSi_2_)

A suspension of 15 mg **12** (29 μmol), 26 mg **4** (59 μmol), and molecular sieves 4 Å in 0.2 cm^3^ dry CH_2_Cl_2_ was stirred for 1 h at rt under Ar. Then, a 0.45 M solution of 1*H*-tetrazole in acetonitrile (131 mm^3^, 59 μmol) was added, and stirring was continued for 2 h. The reaction mixture was cooled to −78 °C, and 10 mg *m*CPBA (59 μmol) was added. After 1 h, the reaction was quenched by addition of 10 mm^3^ triethylamine and warmed up to rt. The suspension was diluted with CH_2_Cl_2_ and washed with saturated aqueous NaHCO_3_ and brine. The organic layer was dried (Na_2_SO_4_) and concentrated. The residue was purified by HPLC (YMC-Pack-SiI-06, hexane/EtOAc = 2/1 → 1/2) which gave 8.2 mg (32%) **13** as diastereoisomeric mixture. *R*_f_ = 0.45 (hexane/EtOAc = 1/1); ^1^H NMR (600 MHz, CDCl_3_) for major isomer a: *δ* = 7.38–7.28 (m, 10H, H^Ar^), 5.38 (bs, 1H, NH), 5.19 (dd, 1H, *J* = 1.9, *J* = 3.5 Hz, H-2), 5.10–5.08 (m, 4H, 2 × C*H*_2_Ph), 4.65 (br s, 1H, H-1), 4.63 (ddd, 1H, *J* = 9.5, *J*_P,3_ = 7.6 Hz, H-3), 4.35 (dt, 1H, *J* = 8.9, *J* = 1.5 Hz, H-6), 4.13 (t, 1H, *J* = 9.5 Hz, H-4), 4.13–4.09 (m, 1H, PO*CH*_*2*_CH_2_), 4.07–4.02 (m, 1H, PO*CH*_*2*_CH_2_), 4.03 (dd, 1H, *J* = 12.0, *J* = 8.7 Hz, H-7a), 3.85 (br d, 1H, *J* = 11.9 Hz, H-7b), 3.51 (br d, 1H, *J* = 8.9 Hz, H-5), 3.48–3.39 (m, 2H, NCH_2_ *CH*_*2*_), 3.28 (s, 3H, OCH_3_), 2.01 (s, 3H, OAc), 1.13–0.86 (m, 28H, TIPDS) ppm; ^31^P NMR (243 MHz, CDCl_3_): *δ* = 0.52 ppm; ^1^H NMR (600 MHz, CDCl_3_) for minor isomer b: *δ* = 7.41–7.28 (m, 10H, H^Ar^), 5.28 (bs, 1H, NH), 5.16 (dd, 1H, *J* = 1.9, *J* = 3.5 Hz, H-2), 5.12–5.05 (m, 4H, 2 × CH_2_Ph), 4.65 (d, 1H, *J* = 1.9 Hz, H-1), 4.59 (ddd, 1H, *J* = 3.3, *J*_H,P_ = 7.5, *J* = 9.6 Hz, H-3), 4.36 (br d, 1H, *J* = 8.9 Hz, H-6), 4.15 (t, 1H, *J*_4,5_ = *J*_4,3_ = 9.5 Hz, H-4), 4.11–4.01 (m, 3H, PO*CH*_*2*_CH_2_, H-7a), 3.87 (dd, 1H, *J* = 1.05 Hz, *J* = 12.15 Hz, H-7b), 3.58 (br s, 1H), 3.50 (dd, 1H, *J* = 9.5, *J* = 1.6 Hz, H-5), 3.42–3.36 (br s, 2H, N*CH*_*2*_), 3.29 (s, 3H, OCH_3_), 2.02 (s, 3H, OAc), 1.34–0.93 (m, 28H, TIPDS) ppm; ^31^P NMR (243 MHz, CDCl_3_): *δ* = 0.45 ppm; ^13^C NMR (150 MHz, CDCl_3_): *δ* = 170.1 (C=O), 156.4 (NC=O), 136.47; 135.49, 135.45, 128.81, 128.75, 128.7, 128.6, 128.5, 128.2, 128.11, 128.08, 128.03, 128.00, 127.5 (C^Ar^), 54.91 (OCH_3_); additional signals for isomer a: 98.3 (C-1), 77.6 (*J*_C,P_ = 6.0 Hz, C-3), 73.5 (C-6), 72.51 (C-5), 70.1 (CH_2_Ph), 70.0 (C-2), 68.0 (C-7), 67.4 (*J*_C,P_ = 6.2 Hz, CH_2_Ph), 66.1 (C-4) and 41.3 (N*C*H_2_) ppm; additional signals for isomer b: 98.2 (C-1), 77.5 (*J*_C,P_ = 6.3 Hz, C-3), 73.6 (C-6), 72.5 (C-5), 70.1 (CH_2_Ph), 70.0 (C-2), 68.0 (C-7), 67.4 (*J*_C,P_ = 6.2 Hz, CH_2_Ph), 66.1 (C-4), 41.2 (N*C*H_2_) ppm; HRMS (ESI): *m/z* calcd for C_39_H_62_NO_14_PSi_2_ + H^+^ ([M+H]^+^) 856.3519, found 856.3528.

### Methyl 2-*O*-acetyl-3-*O*-[benzyl-(2-*N*-benzyloxycarbonylaminoethyl)phosphono]-l-glycero-α-d-manno-heptopyranoside (**14**, C_27_H_36_NO_13_P)

A solution of 12 mg **13** (14 μmol) in 0.2 cm^3^ CH_2_Cl_2_ was cooled to ice-bath temperature followed by the addition of 38 mg TREAT (0.234 mmol). The solution was stirred for 2 h at rt, a second portion of TREAT (38 mg, 0.234 mmol) was added, and stirring was continued for 17 h. Dowex AG 1X8 anion-exchange resin (HCO_3_^−^-form) was added, the resin was filtered off, and the filtrate was concentrated. The residue was subjected to flash chromatography (hexane/EtOAc = 1/7 → EtOAc → EtOAc/EtOH = 9/1) which gave **14** (6 mg, 70%) as syrup. *R*_f_ = 0.35 (EtOAc/EtOH = 9/1); ^1^H NMR (600 MHz, CDCl_3_): *δ* = 7.39–7.29 (m, 10H, H^Ar^), 5.42 (br s, ~0.5H, NH-a), 5.34 (br s, ~0.5H, NH-b), 5.24 (dd, ~0.5H, *J* = 1.5, *J* = 3.5 Hz, H-2b), 5.20–5.18 (m, 0.5H, H-2a), 5.11–5.06 (m, 4H, 2 × CH_2_Ph), 4.65 (m, 1H, H-1a,b), 4.63 (ddd, 1H, *J* = 3.7, *J*_3,P_ = 7.6, *J* = 9.4 Hz, H-3a,b), 4.12 (t, 1H, *J*_4,5_ = *J*_4,3_ = 9.8 Hz, H-4a,b), 4.14–4.02 (m, 3H, H-6a,b, OP*CH*_*2*_CH_2_), 3.85–3.78 (m, 1H, H-7a,b), 3.73–3.68 (m, 1H, H-7′a,b), 3.63–3.58 (m, 1H, H-5), 3.45–3.67 (m, 2H, N*CH*_*2*_), 3.34 (s, 1.7H, OCH_3_-a), 3.32 (s, 1.3H, OCH_3_-b), 2.06 and 2.05 (s, 3H, OAc-a,b) ppm; ^13^C NMR (150 MHz, CDCl_3_, selected HSQC data): *δ* = 128.9, 128.7, 128.5, 128.0 (C^Ar^), 98.6 (C-1), 77.3 (C-3), 72.5 (C-5), 70.2 (CH_2_Ph), 70.0, 69.8 (C-2), 69.9 (C-6), 67.4 (PO*CH*_*2*_CH_2_), 66.9 (CH_2_Ph), 66.3 (C-4), 64.4 (C-7), 55.2 (OCH_3_), 41.5 (N*CH*_*2*_), 20.6 (OAc) ppm; ^31^P NMR (243 MHz, CDCl_3_): *δ* = 0.37, 0.22 ppm; HRMS (ESI): *m/z* calcd for C_27_H_36_NO_13_P + H^+^ ([M+H]^+^) 614.1997, found 614.1995.

### Methyl 2-*O*-acetyl-3-*O*-(2-aminoethylphosphono)-l-glycero-α-d-manno-heptopyranoside (**15**, C_12_H_24_NO_11_P)

A suspension of 9 mg **14** (0.015 μmol), 4 mg Pd–C (10%) in 0.5 cm^3^ dry methanol, and 5 mm^3^ acetic acid was hydrogenated at atmospheric pressure for 22 h at rt. The catalyst was removed by filtration over Celite®, and the filtrate was concentrated to give 5.3 mg (93%) **15** as syrup. *R*_f_ = 0.51 (MeOH/CHCl_3_/H_2_O = 10/10/3); [*α*]_D_^20^ = +23.2° cm^2^ g^−1^ (*c* = 0.5, MeOH); ^1^H NMR (600 MHz, MeOD): *δ* = 5.22 (dd, 1H, *J* = 3.6, *J* = 1.7 Hz, H-2), 4.69 (d, 1H, *J* = 1.7 Hz, H-1), 4.47–4.42 (dt, 1H, *J*_2,3_ = 3.6, *J*_3,P_ = *J*_4,3_ = 9.3 Hz, H-3), 4.11–3.99 (m, 4H, H-4, H-6, PO*CH*_*2*_CH_2_), 3.68 (dd, 1H, *J* = 10.7, *J* = 6.8 Hz, H-7a), 3.65–3.62 (m, 2H, H-5, H-7b), 3.37 (s, 3H, OCH_3_), 3.16–3.11 (m. 2H, POCH_2_ *CH*_*2*_), 2.09 (s, 3H, OAc) ppm; ^13^C (150 MHz, MeOD): *δ* = 172.2 (C=O), 99.9 (C-1), 75.6 (d, ^2^ *J*_C-P_ = 5.7 Hz, C-3), 72.7 (d, ^3^ *J*_C-P_ = 2.1 Hz, C-2), 72.5 (C-5), 70.35 (C-6), 67.2 (d, ^3^ *J*_C-P_ = 4.4 Hz, C-4), 64.15 (C-7), 63.2 (d,^2^ *J*_C-P_ = 5.5 Hz, PO*CH*_*2*_CH_2_), 55.4 (OCH_3_), 41.7 (d, ^3^ *J*_C-P_ = 6.6 Hz, N*CH*_*2*_), 20.9 (OAc) ppm; ^31^P NMR (243 MHz, CDCl_3_): *δ* = 0.51 ppm; HRMS (ESI): *m/z* calcd for C_12_H_24_NO_11_P + H^+^ ([M+H]^+^) 390.1160, found 390.1168.

### Methyl 3-*O*-(2-aminoethylphosphono)-l-glycero-α-d-manno-heptopyranoside (**16**, C_10_H_22_NO_10_P)

A solution of 5 mg **15** (0.013 mmol) in 0.5 cm^3^ MeOH/TEA/H_2_O = 8/1/1 was stirred for 3 days at rt. Then, 40 mm^3^ water were added, and stirring was continued for 4 days. The solution was concentrated, and the residue was purified on Sephadex G-10 (H_2_O/EtOH = 95/5) to give 2.8 mg **16** (63%) after lyophilization. *R*_f_ = 0.32 (MeOH/CHCl_3_/H_2_O = 5/4/1); [*α*]_D_^20^ = +18.2° cm^2^ g^−1^ (*c* = 0.28, H_2_O); ^1^H NMR (600 MHz, D_2_O): *δ* = 4.74 (d, 1H, *J* = 1.5 Hz, H-1), 4.21 (ddd, 1H, *J* = 3.4, *J* = 9.8, *J*_3,P_ = 8.5 Hz, H-3), 4.14–4.10 (m, 2H, PO*CH*_*2*_CH_2_), 4.09 (dd, 1H, H-2), 4.02 (m, 1H, *J* = 1.5, *J* = 5.5, *J* = 7.5 Hz, H-6), 3.94 (t, 1H, *J* = 9.8 Hz, H-4), 3.72 (dd, 1H, *J* = 11.3 Hz, H-7a), 3.69 (dd, 1H, H-7b), 3.59 (dd, 1H, H-5), 3.35 (s, 3H, OMe), 3.25 (t, 2H, *J* = 5.0 Hz, C*H*_*2*_N) ppm; ^13^C NMR data (150 MHz, D_2_O): see Table 1; ^31^P NMR (243 MHz, D_2_O): *δ* = −0.29 ppm; HRMS (ESI): *m/z* calcd for C_10_H_22_NO_10_P + K^+^ ([M+K]^+^) 386.0614, found 386.0613.
